# Retrospective evaluation of the agreement between thoracic point-of-care ultrasound and thoracic radiographs in cats with recent trauma: 111 cats

**DOI:** 10.3389/fvets.2024.1376004

**Published:** 2024-06-26

**Authors:** Pierre-André Vidal, Søren R. Boysen, Julie Fordellone, Alexandra Nectoux, Bernard Allaouchiche, Céline Pouzot-Nevoret

**Affiliations:** ^1^Intensive Care Unit (SIAMU), Université de Lyon, VetAgro Sup, Marcy l’Etoile, France; ^2^APCSe, Université de Lyon, VetAgro Sup, Marcy l'Étoile, France; ^3^Faculty of Veterinary Medicine, University of Calgary, Calgary, AB, Canada; ^4^Hospices Civils de Lyon, Centre Hospitalier Lyon-Sud, Service de Réanimation, Pierre-Bénite, France

**Keywords:** cats, trauma, point-of-care ultrasound, pulmonary contusions, pneumothorax, pleural effusion, lung ultrasound

## Abstract

**Introduction:**

Motor vehicular trauma, bite wounds, high-rise syndrome, and trauma of unknown origin are common reasons cats present to the emergency service. In small animals, thoracic injuries are often associated with trauma. The objective of this retrospective study was to evaluate limits of agreement (LOA) between thoracic point-of-care ultrasound (thoracic POCUS) and thoracic radiography (TXR), and to correlate thoracic POCUS findings to animal trauma triage (ATT) scores and subscores in a population of cats suffering from recent trauma.

**Methods:**

Cats that had thoracic POCUS and TXR performed within 24 h of admission for suspected/witnessed trauma were retrospectively included. Thoracic POCUS and TXR findings were assessed as “positive” or “negative” based on the presence or absence of injuries. Cats positive on thoracic POCUS and TXR were assigned 1 to 5 tentative diagnoses: pulmonary contusions/hemorrhage, pneumothorax, pleural effusion, pericardial effusion, and diaphragmatic hernia. When available ATT scores were calculated. To express LOA between the two imaging modalities a kappa coefficient and 95% CI were calculated. Interpretation of kappa was based on Cohen values.

**Results:**

One hundred and eleven cats were included. 83/111 (74.4%) cats were assessed as positive based on thoracic POCUS and/or TXR. Pulmonary contusion was the most frequent diagnosis. The LOA between thoracic POCUS and TXR were moderate for all combined injuries, moderate for pulmonary contusions/hemorrhage, pneumothorax, diaphragmatic hernia, and fair for pleural effusion. Cats with positive thoracic POCUS had significantly higher median ATT scores and respiratory subscores compared to negative thoracic POCUS cats.

**Discussion:**

The frequency of detecting intrathoracic lesions in cats was similar between thoracic POCUS and TXR with fair to moderate LOA, suggesting thoracic POCUS is useful in cats suffering from trauma. Thoracic POCUS may be more beneficial in cats with higher ATT scores, particularly the respiratory score.

## 1 Introduction

Trauma is a common cause of mortality in cats that often results from motor vehicular trauma, bite wounds, high-rise syndrome, and unknown causes (1–3). In small animals, thoracic trauma is present in 39–60% of trauma cases (3, 4), with pneumothorax, lung contusions, pleural effusion, diaphragmatic hernia, and/or rib fractures being the most common thoracic injuries reported (1, 3–7). These lesions are not always evident on physical examination alone, stressing the importance of additional diagnostic modalities, particularly thoracic imaging (3).

Computed tomography (CT) is considered the reference standard to assess intrathoracic injury. However it often requires sedation or anesthesia, transport of the patient, may be cost prohibitive for owners and involves risk of patient and staff exposure to ionizing radiation (8). For these reasons, radiography is arguably the most widely used imaging modality to assess patients for thoracic injury in veterinary medicine, but has similar limitations to CT. Most recently, point-of-care ultrasound (POCUS) has gained acceptance in small animal medicine as it is relatively inexpensive, minimally invasive, radiation sparing, and can be performed patient-side with minimal stress, which is particularly important for low stress handling in cats. Point-of-care ultrasound is widely used and well described in human and canine trauma patients. Moreover, in human medicine, thoracic POCUS is more sensitive than TXR for the detection of many trauma related thoracic injuries, including lung contusions, pneumothorax, rib fractures and pleural effusion (9–12).

Four studies in small animals have compared POCUS to TXR, CT, and/or thoracocentesis for detection of trauma induced thoracic injuries. These studies found thoracic POCUS had good limits of agreement (LOA) for pulmonary contusions, slight to moderate LOA for pneumothorax and fair to moderate LOA for pleural effusion when compared to other diagnostic modalities (13–15). Unfortunately, although cats were sometimes enrolled with dogs, the combined number of cats included in any of these studies was small (*n* = 9) (5, 13–15).

In humans the likelihood of detecting thoracic injury correlates to the injury severity score. Several injury severity scores have been developed in small animals. In cats, the Animal Trauma Triage (ATT) score is probably the most widely applied and studied (2, 16–19). Several studies have shown a correlation between the ATT score and mortality in both dogs and cats (2, 16, 18–25). More recently, the American College of Veterinary Emergency and Critical Care Veterinary Committee on Trauma has also validated the use of the ATT score in a large population of cats (19). The ATT score is based on the evaluation of six independent equally weighted components: perfusion, cardiac, respiratory, eye/muscle/integument, skeletal, and neurologic, and is scored from 0 to 3 (0 for no or slight injuries and 3 for the most severe injuries) (17). No study has correlated the injury severity of trauma in cats with the likelihood of detecting pathology on thoracic POCUS.

The objectives of this study were to evaluate LOA between thoracic POCUS and TXR in a large population of cats with a recent history of trauma and to correlate thoracic POCUS findings to the ATT score. We hypothesized that the overall LOA between thoracic POCUS and TXR will be good for pulmonary contusions, fair to moderate for pleural effusion, slight to moderate for pneumothorax and moderate for all combined injuries. Furthermore, we hypothesized there will be a significant correlation between thoracic POCUS and the ATT score.

## 2 Materials and methods

This retrospective clinical study enrolled cats presented to the intensive care unit (ICU) of VetAgro Sup (SIAMU) with suspected or witnessed trauma between February 2014 and April 2021.

A key word search of the electronic medical data base was performed using the terms “polytrauma,” “trauma,” “hit by car,” “fall,” “jump,” “high rise syndrome” and “bite.” Cats were included if thoracic POCUS and TXR were performed within 24 h of admission. Cats were excluded if thoracic POCUS or TXR findings were not recorded, if thoracocentesis was performed between imaging examinations, or if there was a delay of >24 h between imaging modalities.

The ICU admission date, breed, sex, age, weight, cause of trauma, and the approximate time between trauma and admission to the ICU were recorded. Vital signs including heart rate, respiratory rate, pulse quality, capillary refill time, mucous membrane color, respiratory effort and temperature were also recorded when available. When possible, the ATT score and subscores (perfusion, cardiac, respiratory, eye/muscle/integument, skeletal and neurologic) were calculated (16, 17). Finally, thoracocentesis and outcome (survival/dead) were also recorded.

Thoracic POCUS and TXR were categorically classified as positive or negative based on the presence or absence of detectable thoracic injuries. The following criteria, obtained from the medical records, was used to make a sonographic diagnosis of (1) pulmonary contusions: more than 3 B-lines/coalescent B-lines/pulmonary contusions/and or pulmonary hemorrhage, (2) pneumothorax: absence of lung sliding (glide sign) and/or pneumothorax, (3) pleural effusion: presence of anechoic fluid in the pleural space and or pleural effusion, (4) diaphragmatic hernia: presence of abdominal organs within the thoracic cavity and/or diaphragmatic hernia, and (5) pericardial effusion: anechoic fluid in the pericardial space and/or pericardial effusion. Radiology reports, written by board-certified radiologists, were reviewed to determine the final radiographic diagnosis. The diagnosis of several concomitant lesions was possible for both thoracic POCUS and TXR. Thoracic POCUS examinations were performed and interpreted within the ICU by house officers which included small animal rotating interns, emergency and critical care (ECC) interns, ECC residents and ECC specialists. In all cases, thoracic POCUS was performed on admission and TXR was performed after cats were sufficiently stable to allow radiographs to be safely obtained.

The thoracic POCUS exam was based on the 2008 TFAST protocol and included bilateral chest tube sites (CTS), bilateral pericardial site (PCS) and the diaphragmatico-hepatic site, with patients positioned in either lateral or sternal recumbency, or a standing position (26). However, this protocol was adapted by the attending clinician based on clinical patient assessment and the presence of life-threatening injuries which mandated immediate medical intervention. A microconvex ultrasound transducer with a frequency of 5–8 MHz (convexe SonoSite Edge II, Vet, FUJIFILM, Montigny Le Bretonneux, France) was used for all sonographic scans.

To determine the LOA between thoracic POCUS and TXR a Kappa coefficient and 95% CI were calculated using a commercial statistical software program (Graphpad–Dotmatics company). Statistical significance was set at *p* ≤ 0.05. Interpretation of kappa was based on Cohen values (27): a Kappa value greater than or equal to 0.81 was deemed to have the best agreement, between 0.61 and 0.80 was characterized as substantial agreement, between 0.41 and 0.60 as moderate agreement, between 0.21 and 0.40 as fair agreement, and between 0.00 and 0.20 as slight agreement. A Kappa <0 was characterized as no agreement. Descriptive statistics were calculated for signalment and presentation of variables. Categorical variables were expressed as frequencies or percentages. Quantitative parameters were expressed as mean and standard deviation (SD, normally distributed) or median and range (non-normally distributed). Normality was tested using the Shapiro–Wilk test. A Chi-square or Fisher’s exact test were used to compare categorical data. A Mann Whitney test was used to compare ATT score and thoracic POCUS findings and to compare the survival rate with the ATT score.

## 3 Results

One hundred and twenty-two cases were included for review. Of these 122 cases, 11 cats were excluded; 6 because thoracocentesis was performed between thoracic POCUS and TXR, 3 because there was a lack of information in the record regarding thoracic POCUS findings, and 2 because a diagnosis of diaphragmatic hernia was based on TXR obtained by the referring veterinarian. Of the 111 remaining cats, 54 (48.6%) presented after witnessed or suspected motor vehicular trauma, 53 (47.8%) for high rise syndrome and 4 (3.6%) for canine induced bite wounds.

Mean age was 2.8 ± 2.3 years. There were 25 spayed females, 17 sexually intact females, 47 castrated males, and 22 sexually intact males. Represented breeds included domestic short hair (*n* = 97), four Siamese, three Bengal, two Main Coon, one British, one Chartreux, one Birman, one Norwegian Forest Cat and one Turkish Angora. Ninety-five cats (85.5%) were discharged, 11 cats (10.0%) were euthanized, and 5 cats (4.5%) died during hospitalization.

Thoracic injury was present on thoracic POCUS and/or TXR in 83/111 (74.4%) cats with 56/111 cats (50.4%) having thoracic injury detectable on both thoracic POCUS and TXR. Pulmonary contusions and pneumothorax were the most frequent injuries detected on thoracic POCUS and TXR. In the current study, no cats had pericardial effusion (Table 1).

**Table 1:** Frequency of thoracic lesions found on thoracic point-of-care ultrasound (thoracic POCUS), thoracic radiography (TXR) and simultaneously on both modalities (POCUS and TXR) in a population of 111 cats presented for trauma.

	Thoracic POCUS	TXR	POCUS and TXR	*P*-value*
All lesions combined	63% (70/111)	62% (69/111)	50% (56/111)	1.000
Pulmonary contusions	54% (60/111)	56% (62/111)	41% (46/111)	0.8927
Pneumothorax	17% (19/111)	26% (29/111)	13% (15/111)	0.1418
Pleural effusion	3% (3/111)	14% (16/111)	3% (3/111)	0.003
Diaphragmatic hernia	3% (3/111)	6% (7/111)	3% (3/111)	0.3324
Pericardial effusion	0% (0/111)	0% (0/111)	0% (0/111)	1.00

All lesions combined includes cats that had more than one lesion. *Based on fishers exact two tailed contingency table analysis.

The LOA between thoracic POCUS and TXR for the various thoracic lesions are reported in Table 2, with all agreements ranging from fair to moderate. Cats with and without signs of respiratory distress (*n* = 40 and 71, respectively) were subanalyzed to compare the overall LOA between POCUS and TXR for all lesions combined (Table 3). Samples size was too small in the subcategorized cats to compare values for individual pathologies.

**Table 2:** Limit of agreement between thoracic point-of-care ultrasound (thoracic POCUS) and thoracic radiography (TXR) in a population of 111 cats presented for trauma.

	Kappa	CI 95%	Agreement
All lesions combined	0.48	0.31–0.65	Moderate
Pulmonary contusion	0.45	0.28–0.62	Moderate
Pneumothorax	0.53	0.54–0.94	Moderate
Pleural effusion	0.28	0.03–0.54	Fair
Diaphragmatic hernia	0.58	0.22–0.95	Moderate

CI, confidence interval.

**Table 3:** Overall limit of agreement between thoracic point-of-care ultrasound (thoracic POCUS) and thoracic radiography (TXR) in a population of 111 cats presented for trauma with and without signs of respiratory distress.

	Kappa	CI 95%	Agreement
All lesions combined in cats with respiratory distress	0.24	−0.10-0.578	Fair
All lesions combined in cats without respiratory distress	0.55	0.35–0.74	Moderate

CI, confidence interval.

The ATT score was calculated for 106/111 cats with a mean score of 3.66 (SD = 2.47). The ATT score was higher in cats that died (mean of 5.75, SD = 2.79) compared to cats that survived (3.29, SD = 2.22) which was statistically significant (*p* = 0.0008). There was a statistically significant difference between cats that were positive on thoracic POCUS and cats what were negative on thoracic POCUS (4 versus 3; *p* = 0.0428). For ATT subscores, only the respiratory subscore was statistically different between cats that were positive versus cats that were negative on thoracic POCUS (1 versus 0; *p* = 0.0028).

## 4 Discussion

This is the first study to compare thoracic POCUS and TXR in cats presenting for trauma. Results confirm that thoracic POCUS is a valuable diagnostic modality in this study population, identifying injury in 63% of cases. However, the agreement between thoracic POCUS and TXR for all pathologies was only fair to moderate, suggesting the two imaging modalities are complementary in the feline trauma setting. In addition, cats with a positive thoracic POCUS had a significantly higher ATT score, suggesting thoracic POCUS may have greater application in more severely injured patients.

Thoracic injury was present on thoracic POCUS and/or TXR in 74.4% of cats in the current study. This percentage is close to the upper range previously reported in cats (10–90%) (1–3, 5–7, 20, 28, 29). However, due to the retrospective nature of this study and the inclusion/exclusion criteria, not all cases of cats with trauma were included. Therefore, the true frequency of trauma induced thoracic injury is probably lower than reported. Furthermore, the high prevalence of injury reported in the current study is likely due to the large number of cases that presented for high rise syndrome, which is reported to have the highest prevalence of thoracic injury among canine and feline trauma patients (up to 90%) (28). Similar to a prior feline study, pulmonary contusion and pneumothorax were the most frequent lesions identified (29). Given the high occurrence of thoracic injury reported in cats following trauma, thoracic evaluation with imaging should be standard of care, particularly given that cats with thoracic injury do not always show overt clinical signs of respiratory distress (3).

In the current study, pulmonary contusions had moderate LOA between thoracic POCUS and TXR, which is in line with what has been reported in human and canine trauma studies. Numerous human studies demonstrate a high sensitivity and specificity for thoracic POCUS to detect trauma related pulmonary contusions compared to TXR (11, 30). A canine study by Armenise et al. described similar results, demonstrating a more extensive thoracic POCUS protocol (VetFAST-ABCDE) detected pulmonary contusions in 47% of dogs presenting for trauma, compared to only 20% on TXR (15). Another canine study by Dicker et al. concluded that thoracic POCUS had a higher sensitivity (90.5%) and equal specificity (87.5%) compared to TXR (66.7 and 87.5%, respectively) for the diagnosis of pulmonary contusions when using CT as the reference standard (31) Direct comparison between the current and prior canine studies is difficult as lung ultrasound protocols varied between the TFAST protocol used in the current study and more extensive lung ultrasound protocols used in prior studies. It is possible that a higher percentage of pulmonary contusions would have been detected if a larger lung surface scanning protocol was used, which has been suggested by some authors (32).

The LOA between thoracic POCUS and TXR to detect pneumothorax was moderate in the current population of cats, which is similar to mixed results demonstrated in other small animal studies (13–15, 21, 33, 34). One canine study reported TFAST had a sensitivity of only 20% compared to TXR (21), while a second study showed an overall sensitivity of 78.1% and specificity of 93.4% with 7 false positives and 7 false negatives when compared to TXR (14). Comparing a mixed population of cats and dogs to CT in trauma patients, TFAST was shown to have a negative correlation with CT for the diagnosis of pneumothorax (13). A second mixed study population using the Vet BLUE protocol compared to CT in dogs and cats presenting to the emergency service showed that Vet BLUE was only 33% accurate for the diagnosis of pneumothorax (33). Finally, a canine study evaluating a horizontal sliding lung ultrasound protocol using thoracocentesis as the reference standard demonstrated that pleural and lung ultrasound has a higher sensitivity than TXR for detection of pneumothorax (15). There are several factors that likely explain these mixed results. The most widely used sonographic criterion to diagnose pneumothorax in both human and veterinary medicine is the absence of lung sliding. Although not clinically studied in small animals, other sonographic criteria have been described in both human and veterinary medicine to increase the likelihood of correctly ruling in (see-shore sign on M-mode, visualization of lung point, abnormal curtain sign) or ruling out the diagnosis (detection of B-lines, visualization of the lung pulse) (34–36). Because the current study only assessed lung sliding it may have underdiagnosed the presence of pneumothorax. Thoracic POCUS windows assessed may also have contributed to these results. The chest tube site was the most common site assessed for the presence or absence of pneumothorax in the current study, while larger lung surface areas were assessed in other studies (15, 33, 36). Moreover, the position of chest tube site can vary from operator to operator and the most gravity independent site for air to accumulate will vary depending on if the patient is in sternal or lateral recumbency (35). Although patient position was not specifically recorded in the current study, it is possible that assessing the more caudo-dorsal sites when cats were in a sternal or standing position, the widest point of the chest when cats were in a lateral position, and incorporating the curtain sign, may have increased the LOA between imaging modalities (34–36).

The sonographic detection of pleural effusion in the current study was low (3%) and had fair LOA with TXR, which is similar to what has been reported in other small animal studies following trauma (13, 15, 33). A trauma study in cats and dogs identified pleural effusion in 38% of cases using CT, and reported that TFAST had fair to moderate correlation to CT for detection of pleural effusion, depending on the operator performing the sonographic examination (13). Another study comparing TXR and CT in 59 dogs presenting with blunt motor vehicular trauma identified pleural effusion in 12% of cases using CT, compared to only 5% on TXR (37). Finally, a canine study identified pleural effusion in 6% of trauma patients using TFAST (14). The variation in detection of pleural effusion in small animals is likely a result of the imaging modality used, making it difficult to know the true prevalence of pleural effusion and therefore the sensitivity and specificity of thoracic POCUS to identify pleural effusion in cats. Indeed, based on a canine cadaver study and a small case study in dogs, Boysen et al. suggest that probe orientation and location, as well as patient position play an important role in pleural fluid detection. They suggest orientating the probe parallel to the ribs and scanning multiple sites along the ventral sternal border with the patient in a sternal or standing position, or scanning the widest gravity dependent site with the patient in lateral position, may increase the sensitivity and specificity of detecting pleural effusion (36–38). Due to the retrospective nature of the study, the exact probe orientation and location on the thoracic wall cannot be determined and small quantities of fluid may have been missed.

Diaphragmatic hernia is another traumatic injury well described in the veterinary literature (14, 15, 20, 28, 29, 39). Our study showed a moderate LOA between thoracic POCUS and TXR for diaphragmatic hernia, which has not previously been described in cats. However, these results should be interpreted with caution due to the low number of cases with diaphragmatic hernia. In the study by Armenise et al., thoracic POCUS was able to detect one case of diaphragmatic hernia which was not detected on TXR (15). Another study that compared the use of ultrasound with clinical signs of dyspnea and muffled heart sounds on auscultation to diagnose diaphragmatic rupture in dogs and cats compared to radiographs, surgery, or necropsy found the accuracy of ultrasonography was 93%. However, this study was conducted by imaging specialists in a controlled setting, and not by less experienced operators in the emergency setting, as was the situation in the current study (40).

Our study showed that cats with positive thoracic POCUS have significantly higher ATT scores, particularly the respiratory subscore. This is not surprising as the assessment of the respiratory subscore depends on clinical signs, which will be affected by intra-thoracic lesions. This clinical application of POCUS has also been highlighted in the McMurray et al. study in non-trauma patients, showing that the more unstable the patient is, the greater is the probability of finding POCUS lesions (41). Another study that enrolled 50 dogs suffering trauma demonstrated no significant association between ATT score and TFAST. However, only a small proportion of the population showed lesions on TFAST (21). When analyzing cats with and without respiratory distress the LOA between thoracic POCUS and TXR was higher in cats without respiratory distress which is likely explained by the higher number of true negative cases in this subgroup. Although POCUS is generally considered better at ruling in findings, many of the cats presenting without respiratory distress were likely true negative cases, which would tend to increase the LOA for negative findings in both thoracic POCUS and TXR (e.g., POCUS cannot miss lesions if there are no lesions to miss). By contrast, most cases with respiratory distress had pathology detected on thoracic POCUS and/or TXR, resulting in a very small number of cases without detectable pathology, increasing the likelihood that thoracic POCUS and/or TXR will miss some of these lesions. Larger studies using CT as the reference standard are required to corroborate this hypothesis.

This study has several limitations, mainly due to its retrospective nature. The interpretation of recorded data within the medical record required some subjective assessment to draw conclusions. Several operators performed thoracic POCUS with different levels of training and it is possible that lesions may have been missed by less experienced operators. The thoracic POCUS protocol used was initially based on the 2008 Lisciandro TFAST study, but was adapted by the clinician based on the clinical assessment of the patient, and likely evolved over the study duration as newer thoracic POCUS research findings in small animals became available. Although it is not possible to determine due to the retrospective nature, more recent cases enrolled in the study may have included more extensive thoracic POCUS protocols and therefore influenced findings. Another limitation is that the exact time between each imaging modality was not always possible to determine. Although both thoracic POCUS and TXR were performed within 24 h of admission, in some instances, lesions may have progressed, improved or resolved between imaging modalities, depending on the nature of the condition and time between diagnostics (42). Moreover, the results of TXR and thoracic POCUS have never been compared to the reference standard CT. As a result, some lesions may have been misclassified or misdiagnosed, making it impossible to know the true sensitivity, specificity, and accuracy of either modality. Finally, although the LOA between the two modalities was higher in cats without respiratory distress compared to cats with respiratory distress, LOA was not possible to calculate for individual pathologies due to the small sample size. This may have overestimated the LOA for the subcategories of cats with and without respiratory distress, as any finding was considered positive on thoracic POCUS and TXR, and individual discrepancies were not considered for the LOA calculation (e.g., thoracic POCUS may have identified only contusions in one cat and TXR identified pleural effusion in the same cat, but both would be considered positive).

Due to the retrospective nature of the study, it was not possible to determine if radiologists were blinded to the results of thoracic POCUS. It was also not possible to compare the localization of lesions between thoracic POCUS and TXR as the exact site of injury was not recorded during thoracic POCUS examination. Another limitation is that some cats were removed from the study if thoracocentesis was required following thoracic POCUS, and prior to TXR. This may have introduced a bias and decreased the coefficient of agreement for some injuries (e.g., pneumothorax). Finally, the ATT score was calculated retrospectively based on availability of information in the medical record, without specifically including the time the data was collected within a 24-h window. Therefore, these scores may have been different if they had been calculated at the time of admission.

## 5 Conclusion

Thoracic POCUS is a complementary and useful diagnostic imaging modality that can be used to detect trauma induced thoracic injury in cats. A higher ATT score, particularly the respiratory subscore, suggests a higher probability to identifying thoracic lesions, and particular attention should be paid to these cats when it comes to thoracic POCUS. Further prospective studies using CT as the reference standard and comparing different thoracic POCUS protocol is recommended.

## Author’s note

This retrospective study was presented in part as oral abstract at the 2022 EVECC Congress in Ghent.

## Data availability statement

The raw data supporting the conclusions of this article will be made available by the authors, without undue reservation.

## Ethics statement

Ethical approval is not required for studies involving animals in accordance with the local legislation and institutional requirements because only medical records were reviewed retrospectively. Written informed consent was not obtained from the owners for the participation of their animals in this study because ethical review and approval were not required because only medical records were reviewed retrospectively.

## Author contributions

P-AV: Writing – original draft, Writing – review & editing, Conceptualization, Data curation, Formal analysis, Investigation, Methodology, Validation. SB: Writing – review & editing, Conceptualization, Formal analysis, Investigation, Methodology, Project administration, Supervision, Validation. JF: Writing – original draft, Conceptualization, Data curation, Formal analysis, Investigation, Methodology, Validation. AN: Writing – review & editing, Conceptualization, Investigation, Methodology, Supervision, Validation. BA: Writing – review & editing, Conceptualization, Formal analysis, Investigation, Methodology, Project administration, Software, Supervision, Validation, Writing – original draft. CP-N: Writing – review & editing, Conceptualization, Data curation, Formal analysis, Investigation, Methodology, Project administration, Software, Supervision, Validation, Writing – original draft.
